# Methyl (2*Z*)-2-[(2-formyl-3-methyl-1*H*-indol-1-yl)meth­yl]-3-(4-meth­oxy­phen­yl)prop-2-enoate

**DOI:** 10.1107/S1600536814005261

**Published:** 2014-03-15

**Authors:** S. Selvanayagam, B. Sridhar, S. Kathiravan, R. Raghunathan

**Affiliations:** a Department of Physics, Kalasalingam University, Krishnankoil 626 126, India; b Laboratory of X-ray Crystallography, Indian Institute of Chemical Technology, Hyderabad 500 067, India; c Department of Organic Chemistry, University of Madras, Guindy Campus, Chennai 600 025, India

## Abstract

In the title indole derivative, C_22_H_21_NO_4_, the dihedral angle between the benzene and pyrrole rings of indole moiety is 1.8 (1)°. The plane of the 4-meth­oxy­phenyl ring is oriented with a dihedral angle of 60.7 (1)° with respect to the plane of the indole moiety. The mol­ecular packing is stabilized by C—H⋯O hydrogen bonds which form a V-shaped chain arrangement along the *bc* plane of the unit cell. In addition to this, C—H⋯π and π–π inter­actions [centroid–centroid distances = 3.8102 (11) and 3.8803(12) Å], which run along the *b*-axis direction, stabilize the mol­ecular packing.

## Related literature   

For general background to indole derivatives, see: Kaushik *et al.* (2013 ▶); Singh *et al.* (2000 ▶); Andreani *et al.* (2001 ▶); Grinev *et al.* (1984 ▶); Rodriguez *et al.* (1985 ▶). For a related structure, see: Selvanayagam *et al.* (2008 ▶). For the superposition of a related structure, see: Gans & Shalloway (2001 ▶)
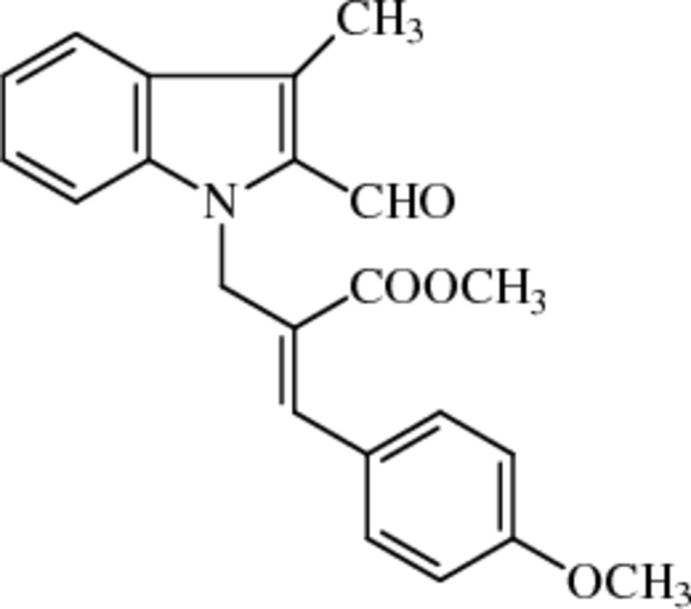


## Experimental   

### 

#### Crystal data  

C_22_H_21_NO_4_
*M* *_r_* = 363.40
Monoclinic, 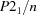

*a* = 12.6009 (13) Å
*b* = 10.7458 (11) Å
*c* = 14.8937 (16) Å
β = 111.954 (2)°
*V* = 1870.5 (3) Å^3^
*Z* = 4
Mo *K*α radiation
μ = 0.09 mm^−1^
*T* = 292 K
0.20 × 0.18 × 0.16 mm

#### Data collection  

Bruker SMART APEX CCD area-detector diffractometer
21494 measured reflections
4462 independent reflections
3214 reflections with *I* > 2σ(*I*)
*R*_int_ = 0.030

#### Refinement  

*R*[*F* ^2^ > 2σ(*F* ^2^)] = 0.052
*wR*(*F* ^2^) = 0.143
*S* = 1.01
4462 reflections
247 parameters
H-atom parameters constrained
Δρ_max_ = 0.21 e Å^−3^
Δρ_min_ = −0.15 e Å^−3^

### 

Data collection: *SMART* (Bruker, 2001 ▶); cell refinement: *SAINT* (Bruker, 2001 ▶); data reduction: *SAINT*; program(s) used to solve structure: *SHELXS97* (Sheldrick, 2008 ▶); program(s) used to refine structure: *SHELXL2013* (Sheldrick, 2008 ▶); molecular graphics: *ORTEP-3 for Windows* (Farrugia, 2012 ▶) and *PLATON* (Spek, 2009 ▶); software used to prepare material for publication: *SHELXL2013* and *PLATON*.

## Supplementary Material

Crystal structure: contains datablock(s) I, global. DOI: 10.1107/S1600536814005261/zq2219sup1.cif

Structure factors: contains datablock(s) I. DOI: 10.1107/S1600536814005261/zq2219Isup2.hkl

Supporting information file. DOI: 10.1107/S1600536814005261/zq2219Isup3.cml

CCDC reference: 990594

Additional supporting information:  crystallographic information; 3D view; checkCIF report

## Figures and Tables

**Table 1:** Hydrogen-bond geometry (Å, °) *Cg*1 and *Cg*2 are the centroids of the N1/C1/C6–C8 and C1–C6 rings, respectively.

*D*—H⋯*A*	*D*—H	H⋯*A*	*D*⋯*A*	*D*—H⋯*A*
C9—H9*A*⋯O1	0.96	2.53	3.033 (3)	113
C14—H14⋯O2	0.93	2.41	2.789 (2)	104
C10—H10*B*⋯O2^i^	0.97	2.51	3.480 (2)	173
C22—H22⋯O2^i^	0.93	2.49	3.409 (3)	171
C17—H17⋯*Cg*1^ii^	0.93	2.76	3.573 (2)	146
C21—H21*A*⋯*Cg*2^ii^	0.96	2.84	3.635 (3)	140

Symmetry codes: (i) 
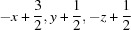
; (ii) 
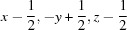
.

**Fig. 1.**
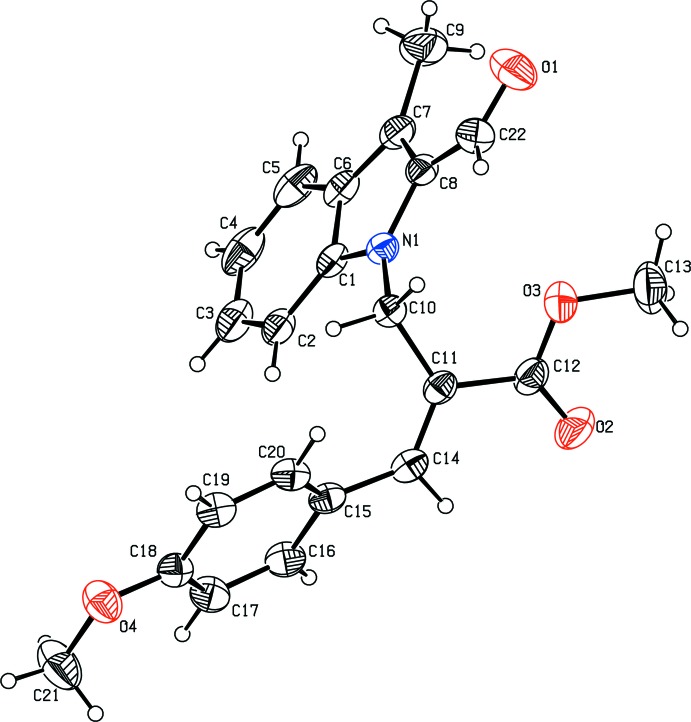
The molecular structure of the title compound, showing the atom-numbering scheme. Displacement ellipsoids are drawn at the 30% probability level.

**Fig. 2.**
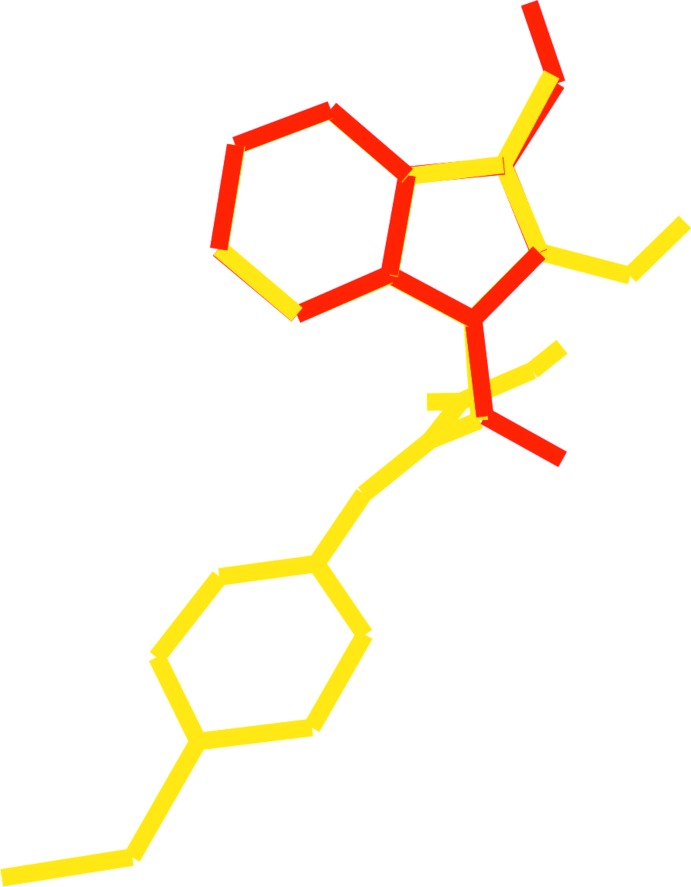
Superposition of (I) (yellow) with the similar reported structure Selvanayagam et al. (2008) (red).

**Fig. 3.**
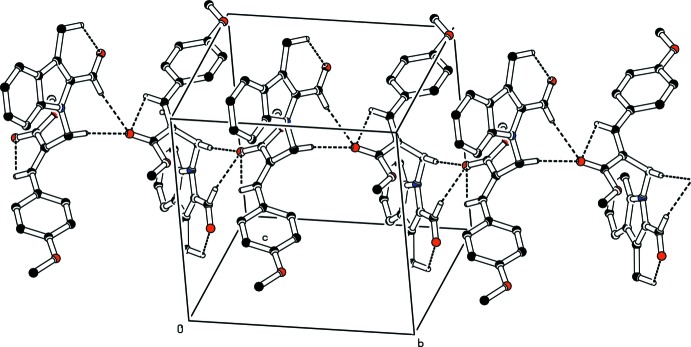
Molecular packing of the title compound, viewed down the c axis; H-bonds are shown as dashed lines·For the sake of clarity, H atoms, not involved in hydrogen bonds, have been omitted

**Fig. 4.**
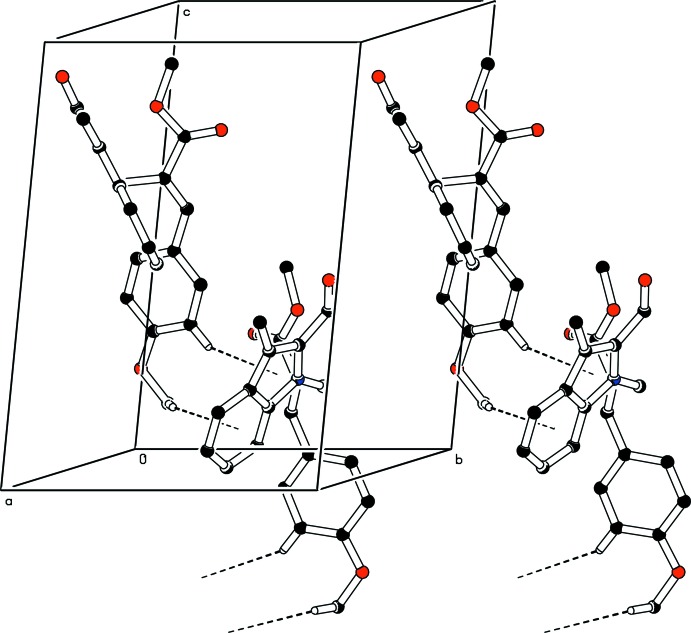
Molecular packing of the title compound, viewed along the a axis; C—H···π interactions are shown as dashed lines·For the sake of clarity, H atoms, not involved in hydrogen bonds, have been omitted

**Fig. 5.**
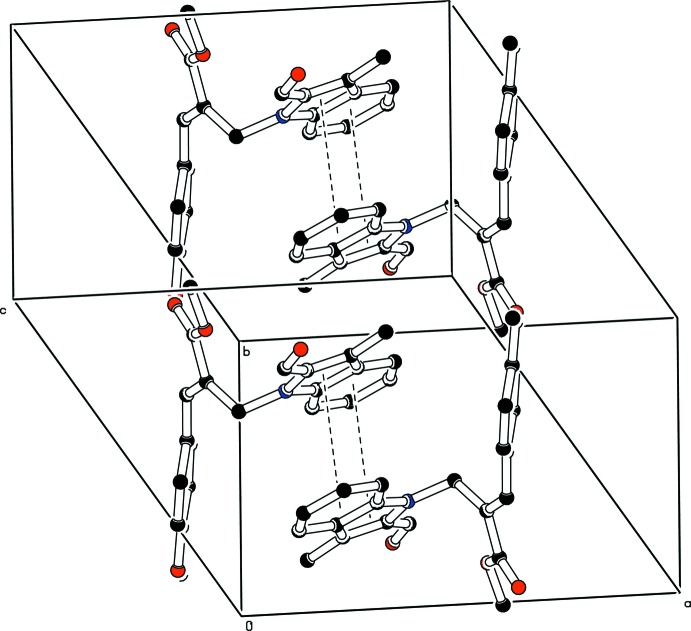
Molecular packing of the title compound, showing π···π interactions. For the sake of clarity, H atoms, not involved in hydrogen bonds, have been omitted

## supplementary crystallographic information

### 1. Comment

Indole is the parent substance of a large number of important compounds that occur in nature with significant biological activity (Kaushik *et al.*, 2013). Indole derivatives exhibit antibacterial, antifungal (Singh *et al.*, 2000), antitumour (Andreani *et al.*, 2001), antidepressant (Grinev *et al.*, 1984) and anti-inflammatory (Rodriguez *et al.*, 1985) activities. In view of that importance, we have undertaken the crystal structure determination of the title compound, and the results are presented here.

The X-ray study confirmed the molecular structure and atomic connectivity for (I), as illustrated in Fig. 1. The geometry of the indole ring system in the present structure is comparable with the related reported structure (Selvanayagam *et al.*, 2008). Fig. 2 shows a superposition of the indole ring system of (I) with this related reported structure, using Qmol (Gans & Shalloway, 2001); the r.m.s. deviation is 0.016 Å.

The sum of the angles at N1 of the indole ring (360°) is in accordance with *sp*^2^ hybridization. The widening of the C14—C15—C20 and N1—C8—C22 bond angles [122.6 (2)° and 122.4 (2)°, respectively] are due to the short contacts H10A···H20 (2.2 Å) and H10B···H22 (2.1 Å).

The indole ring system is planar with a maximum deviation of 0.019 (1) Å for atom C3. The carbaldehyde group atoms (C22 and O1) and methyl atom (C9) deviate 0.111 (1), 0.088 (2) and 0.065 (1) Å, respectively from the best plane of the indole ring. The methoxy group atoms (O4 and C21) deviate -0.015 (1) and -0.040 (1) Å, respectively from the best plane of the methoxy phenyl ring. This ring makes a dihedral angle of 60.7 (1)° with indole ring.

In addition to the van der Waals interactions, the molecular structure is influenced by intramolecular C—H···O interactions (Table 1). In the molecular packing, two C—H···O hydrogen bonds form a V-shaped chain arrangement along '*bc*' plane of the unit cell (Fig. 3). In addition to this weak C—H···π and π···π interactions stabilizes the molecular packing (Fig. 4 and Fig. 5).

### 2. Experimental

POCl_3_ (1 ml) was added drop wise with stirring to DMF (4.25 ml) at 10–20°C over 20 minutes. Then (*E*)-methyl 4-(3-methyl-1*H*-indol-1-yl)-3-(4-methoxy phenyl)but-2-enoate (1 g) in DMF (3 ml) was added slowly with stirring and the mixture was heated. Excess concentrated aqueous solution of NaOAc was added. The mixture was stirred for 30 minutes at 28°C and extracted with AcOEt (3x20ml). The dried (MgSO_4_) extract after removal of solvent furnished a pale yellow oil (1.10 g), which was chromatographed on a silica gel column. Elution with light petroleum-ether/ethyl acetate (3:1) afforded the product in 80% yield. Single crystals of (I) were obtained by slow evaporation of methanol solution of the title compound at room temperature.

### 3. Refinement

H atoms were placed in idealized positions and allowed to ride on their parent atoms, with C—H distances of 0.93–0.96 Å, and *U*_iso_(H) = 1.5*U*_eq_(methyl C) and *U*_iso_(H) = 1.2*U*_eq_ for other C atoms.

### Crystal data

**Table:**

C_22_H_21_NO_4_	*F*(000) = 768
*M**_r_* = 363.40	*D*_x_ = 1.290 Mg m^−^^3^
Monoclinic, *P*2_1_/*n*	Mo *K*α radiation, λ = 0.71073 Å
*a* = 12.6009 (13) Å	Cell parameters from 13428 reflections
*b* = 10.7458 (11) Å	θ = 2.2–27.7°
*c* = 14.8937 (16) Å	µ = 0.09 mm^−^^1^
β = 111.954 (2)°	*T* = 292 K
*V* = 1870.5 (3) Å^3^	Block, colourless
*Z* = 4	0.20 × 0.18 × 0.16 mm

### Data collection

**Table:**

Bruker SMART APEX CCD area-detector diffractometer	*R*_int_ = 0.030
Radiation source: fine-focus sealed tube	θ_max_ = 28.0°, θ_min_ = 1.8°
ω scans	*h* = −16→16
21494 measured reflections	*k* = −14→14
4462 independent reflections	*l* = −19→19
3214 reflections with *I* > 2σ(*I*)	

### Refinement

**Table:**

Refinement on *F*^2^	0 restraints
Least-squares matrix: full	Hydrogen site location: inferred from neighbouring sites
*R*[*F*^2^ > 2σ(*F*^2^)] = 0.052	H-atom parameters constrained
*wR*(*F*^2^) = 0.143	*w* = 1/[σ^2^(*F*_o_^2^) + (0.069*P*)^2^ + 0.3592*P*] where *P* = (*F*_o_^2^ + 2*F*_c_^2^)/3
*S* = 1.01	(Δ/σ)_max_ = 0.001
4462 reflections	Δρ_max_ = 0.21 e Å^−^^3^
247 parameters	Δρ_min_ = −0.15 e Å^−^^3^

### Special details

**Table:**

Geometry. All e.s.d.'s (except the e.s.d. in the dihedral angle between two l.s. planes) are estimated using the full covariance matrix. The cell e.s.d.'s are taken into account individually in the estimation of e.s.d.'s in distances, angles and torsion angles; correlations between e.s.d.'s in cell parameters are only used when they are defined by crystal symmetry. An approximate (isotropic) treatment of cell e.s.d.'s is used for estimating e.s.d.'s involving l.s. planes.

### Fractional atomic coordinates and isotropic or equivalent isotropic displacement parameters (Å^2^)

**Table:**

	*x*	*y*	*z*	*U*_iso_*/*U*_eq_	
O1	1.09513 (15)	0.60014 (19)	0.38553 (11)	0.1024 (6)	
O2	0.73093 (13)	0.23166 (15)	0.23313 (11)	0.0890 (5)	
O3	0.83214 (11)	0.40122 (13)	0.29526 (9)	0.0682 (4)	
O4	0.44555 (12)	0.52878 (12)	−0.32562 (9)	0.0700 (4)	
N1	0.93920 (10)	0.46791 (12)	0.15072 (9)	0.0437 (3)	
C1	0.97938 (13)	0.39988 (13)	0.09250 (12)	0.0453 (4)	
C2	0.92204 (16)	0.35281 (16)	0.00003 (13)	0.0555 (4)	
H2	0.8437	0.3640	−0.0321	0.067*	
C3	0.9859 (2)	0.28912 (17)	−0.04190 (16)	0.0710 (6)	
H3	0.9499	0.2570	−0.1039	0.085*	
C4	1.1026 (2)	0.27130 (18)	0.00578 (19)	0.0789 (7)	
H4	1.1428	0.2267	−0.0247	0.095*	
C5	1.15958 (17)	0.31737 (18)	0.09595 (18)	0.0709 (6)	
H5	1.2379	0.3047	0.1270	0.085*	
C6	1.09796 (14)	0.38477 (15)	0.14165 (13)	0.0534 (4)	
C7	1.12943 (14)	0.44737 (16)	0.23098 (13)	0.0559 (4)	
C8	1.03115 (13)	0.49785 (15)	0.23520 (12)	0.0481 (4)	
C9	1.24838 (17)	0.4581 (3)	0.30555 (18)	0.0886 (7)	
H9A	1.2584	0.5388	0.3352	0.133*	
H9B	1.3028	0.4471	0.2751	0.133*	
H9C	1.2601	0.3952	0.3541	0.133*	
C10	0.81868 (12)	0.50076 (14)	0.12346 (12)	0.0450 (3)	
H10A	0.7900	0.5348	0.0584	0.054*	
H10B	0.8118	0.5646	0.1670	0.054*	
C11	0.74697 (12)	0.39005 (14)	0.12710 (12)	0.0469 (4)	
C12	0.76846 (14)	0.33019 (18)	0.22202 (13)	0.0571 (4)	
C13	0.8478 (2)	0.3602 (3)	0.39105 (15)	0.0955 (8)	
H13A	0.7749	0.3565	0.3976	0.143*	
H13B	0.8967	0.4176	0.4376	0.143*	
H13C	0.8822	0.2791	0.4020	0.143*	
C14	0.66281 (13)	0.34297 (16)	0.05107 (12)	0.0521 (4)	
H14	0.6321	0.2685	0.0621	0.063*	
C15	0.61153 (12)	0.39180 (15)	−0.04761 (12)	0.0483 (4)	
C16	0.57841 (15)	0.31163 (16)	−0.12596 (13)	0.0573 (4)	
H16	0.5927	0.2270	−0.1150	0.069*	
C17	0.52504 (16)	0.35310 (17)	−0.21948 (13)	0.0588 (4)	
H17	0.5059	0.2973	−0.2708	0.071*	
C18	0.49998 (14)	0.47814 (16)	−0.23680 (12)	0.0514 (4)	
C19	0.53108 (14)	0.55973 (15)	−0.15976 (13)	0.0530 (4)	
H19	0.5147	0.6440	−0.1708	0.064*	
C20	0.58585 (13)	0.51745 (15)	−0.06720 (12)	0.0508 (4)	
H20	0.6064	0.5739	−0.0162	0.061*	
C21	0.4105 (3)	0.4468 (2)	−0.40614 (16)	0.0957 (8)	
H21A	0.4760	0.4035	−0.4086	0.144*	
H21B	0.3751	0.4937	−0.4646	0.144*	
H21C	0.3567	0.3877	−0.3996	0.144*	
C22	1.01886 (18)	0.57581 (18)	0.30950 (14)	0.0651 (5)	
H22	0.9471	0.6098	0.2981	0.078*	

### Atomic displacement parameters (Å^2^)

**Table:**

	*U*^11^	*U*^22^	*U*^33^	*U*^12^	*U*^13^	*U*^23^
O1	0.0958 (12)	0.1354 (16)	0.0626 (9)	−0.0236 (11)	0.0141 (8)	−0.0265 (9)
O2	0.0856 (10)	0.0859 (10)	0.0928 (11)	−0.0224 (8)	0.0304 (8)	0.0320 (8)
O3	0.0729 (8)	0.0836 (9)	0.0526 (7)	−0.0043 (7)	0.0284 (6)	0.0071 (6)
O4	0.0881 (9)	0.0592 (8)	0.0563 (8)	0.0043 (7)	0.0197 (7)	−0.0027 (6)
N1	0.0396 (6)	0.0444 (7)	0.0481 (7)	−0.0008 (5)	0.0174 (6)	0.0023 (5)
C1	0.0491 (8)	0.0369 (7)	0.0578 (9)	−0.0016 (6)	0.0290 (7)	0.0064 (7)
C2	0.0657 (10)	0.0494 (9)	0.0614 (10)	−0.0101 (8)	0.0351 (9)	−0.0008 (8)
C3	0.0993 (16)	0.0541 (11)	0.0823 (13)	−0.0183 (10)	0.0601 (12)	−0.0107 (9)
C4	0.1000 (17)	0.0518 (11)	0.1216 (19)	−0.0048 (10)	0.0836 (16)	−0.0062 (12)
C5	0.0616 (11)	0.0558 (11)	0.1150 (17)	0.0047 (9)	0.0556 (12)	0.0115 (11)
C6	0.0482 (9)	0.0452 (9)	0.0750 (12)	0.0008 (7)	0.0324 (8)	0.0135 (8)
C7	0.0436 (8)	0.0562 (10)	0.0655 (11)	−0.0032 (7)	0.0177 (8)	0.0156 (8)
C8	0.0448 (8)	0.0475 (8)	0.0499 (9)	−0.0061 (6)	0.0152 (7)	0.0068 (7)
C9	0.0472 (11)	0.1087 (18)	0.0958 (17)	−0.0028 (11)	0.0107 (10)	0.0148 (14)
C10	0.0420 (8)	0.0429 (8)	0.0502 (8)	0.0020 (6)	0.0173 (7)	0.0027 (7)
C11	0.0396 (8)	0.0479 (9)	0.0575 (10)	0.0023 (6)	0.0232 (7)	0.0046 (7)
C12	0.0454 (9)	0.0649 (11)	0.0660 (11)	0.0019 (8)	0.0266 (8)	0.0137 (9)
C13	0.0927 (16)	0.144 (2)	0.0604 (13)	0.0094 (15)	0.0402 (12)	0.0229 (14)
C14	0.0445 (8)	0.0483 (9)	0.0676 (11)	−0.0025 (7)	0.0255 (8)	0.0015 (8)
C15	0.0373 (7)	0.0489 (9)	0.0602 (10)	−0.0023 (6)	0.0201 (7)	−0.0044 (7)
C16	0.0573 (10)	0.0437 (9)	0.0696 (12)	−0.0010 (7)	0.0221 (9)	−0.0051 (8)
C17	0.0648 (11)	0.0505 (10)	0.0601 (11)	−0.0014 (8)	0.0220 (9)	−0.0133 (8)
C18	0.0484 (9)	0.0522 (9)	0.0554 (10)	0.0004 (7)	0.0215 (8)	−0.0047 (7)
C19	0.0497 (9)	0.0432 (8)	0.0661 (11)	0.0039 (7)	0.0216 (8)	−0.0042 (8)
C20	0.0442 (8)	0.0478 (9)	0.0598 (10)	0.0007 (7)	0.0186 (7)	−0.0122 (7)
C21	0.138 (2)	0.0762 (15)	0.0564 (12)	0.0015 (14)	0.0173 (13)	−0.0096 (11)
C22	0.0671 (11)	0.0654 (11)	0.0594 (11)	−0.0121 (9)	0.0198 (9)	−0.0061 (9)

### Geometric parameters (Å, º)

**Table:**

O1—C22	1.209 (2)	C9—H9C	0.9600
O2—C12	1.196 (2)	C10—C11	1.507 (2)
O3—C12	1.327 (2)	C10—H10A	0.9700
O3—C13	1.435 (2)	C10—H10B	0.9700
O4—C18	1.356 (2)	C11—C14	1.328 (2)
O4—C21	1.419 (2)	C11—C12	1.483 (2)
N1—C1	1.3678 (19)	C13—H13A	0.9600
N1—C8	1.3926 (19)	C13—H13B	0.9600
N1—C10	1.4610 (18)	C13—H13C	0.9600
C1—C2	1.390 (2)	C14—C15	1.464 (2)
C1—C6	1.406 (2)	C14—H14	0.9300
C2—C3	1.371 (3)	C15—C16	1.383 (2)
C2—H2	0.9300	C15—C20	1.394 (2)
C3—C4	1.387 (3)	C16—C17	1.375 (2)
C3—H3	0.9300	C16—H16	0.9300
C4—C5	1.358 (3)	C17—C18	1.382 (2)
C4—H4	0.9300	C17—H17	0.9300
C5—C6	1.409 (3)	C18—C19	1.379 (2)
C5—H5	0.9300	C19—C20	1.368 (2)
C6—C7	1.409 (3)	C19—H19	0.9300
C7—C8	1.375 (2)	C20—H20	0.9300
C7—C9	1.499 (3)	C21—H21A	0.9600
C8—C22	1.442 (3)	C21—H21B	0.9600
C9—H9A	0.9600	C21—H21C	0.9600
C9—H9B	0.9600	C22—H22	0.9300
			
C12—O3—C13	117.18 (17)	C14—C11—C10	124.63 (15)
C18—O4—C21	117.36 (15)	C12—C11—C10	118.58 (14)
C1—N1—C8	108.43 (13)	O2—C12—O3	122.96 (17)
C1—N1—C10	123.07 (13)	O2—C12—C11	125.11 (18)
C8—N1—C10	128.50 (13)	O3—C12—C11	111.89 (15)
N1—C1—C2	130.12 (15)	O3—C13—H13A	109.5
N1—C1—C6	107.75 (14)	O3—C13—H13B	109.5
C2—C1—C6	122.11 (15)	H13A—C13—H13B	109.5
C3—C2—C1	117.19 (18)	O3—C13—H13C	109.5
C3—C2—H2	121.4	H13A—C13—H13C	109.5
C1—C2—H2	121.4	H13B—C13—H13C	109.5
C2—C3—C4	121.7 (2)	C11—C14—C15	129.15 (15)
C2—C3—H3	119.1	C11—C14—H14	115.4
C4—C3—H3	119.1	C15—C14—H14	115.4
C5—C4—C3	121.58 (18)	C16—C15—C20	116.97 (16)
C5—C4—H4	119.2	C16—C15—C14	120.29 (15)
C3—C4—H4	119.2	C20—C15—C14	122.56 (15)
C4—C5—C6	118.82 (19)	C17—C16—C15	122.12 (16)
C4—C5—H5	120.6	C17—C16—H16	118.9
C6—C5—H5	120.6	C15—C16—H16	118.9
C1—C6—C7	107.79 (14)	C16—C17—C18	119.69 (16)
C1—C6—C5	118.56 (18)	C16—C17—H17	120.2
C7—C6—C5	133.63 (18)	C18—C17—H17	120.2
C8—C7—C6	106.93 (14)	O4—C18—C19	116.06 (15)
C8—C7—C9	127.16 (19)	O4—C18—C17	124.70 (15)
C6—C7—C9	125.90 (18)	C19—C18—C17	119.24 (16)
C7—C8—N1	109.09 (15)	C20—C19—C18	120.44 (16)
C7—C8—C22	128.42 (16)	C20—C19—H19	119.8
N1—C8—C22	122.41 (15)	C18—C19—H19	119.8
C7—C9—H9A	109.5	C19—C20—C15	121.53 (15)
C7—C9—H9B	109.5	C19—C20—H20	119.2
H9A—C9—H9B	109.5	C15—C20—H20	119.2
C7—C9—H9C	109.5	O4—C21—H21A	109.5
H9A—C9—H9C	109.5	O4—C21—H21B	109.5
H9B—C9—H9C	109.5	H21A—C21—H21B	109.5
N1—C10—C11	111.97 (12)	O4—C21—H21C	109.5
N1—C10—H10A	109.2	H21A—C21—H21C	109.5
C11—C10—H10A	109.2	H21B—C21—H21C	109.5
N1—C10—H10B	109.2	O1—C22—C8	124.7 (2)
C11—C10—H10B	109.2	O1—C22—H22	117.7
H10A—C10—H10B	107.9	C8—C22—H22	117.7
C14—C11—C12	116.77 (15)		
			
C8—N1—C1—C2	−177.30 (15)	C8—N1—C10—C11	−108.02 (17)
C10—N1—C1—C2	2.3 (2)	N1—C10—C11—C14	−117.11 (17)
C8—N1—C1—C6	1.06 (16)	N1—C10—C11—C12	64.62 (18)
C10—N1—C1—C6	−179.33 (12)	C13—O3—C12—O2	−5.0 (3)
N1—C1—C2—C3	178.78 (15)	C13—O3—C12—C11	172.89 (16)
C6—C1—C2—C3	0.6 (2)	C14—C11—C12—O2	13.9 (3)
C1—C2—C3—C4	0.4 (3)	C10—C11—C12—O2	−167.73 (18)
C2—C3—C4—C5	−0.8 (3)	C14—C11—C12—O3	−163.96 (15)
C3—C4—C5—C6	0.0 (3)	C10—C11—C12—O3	14.4 (2)
N1—C1—C6—C7	−0.97 (17)	C12—C11—C14—C15	170.52 (15)
C2—C1—C6—C7	177.54 (14)	C10—C11—C14—C15	−7.8 (3)
N1—C1—C6—C5	−179.87 (14)	C11—C14—C15—C16	142.41 (18)
C2—C1—C6—C5	−1.4 (2)	C11—C14—C15—C20	−42.6 (2)
C4—C5—C6—C1	1.0 (3)	C20—C15—C16—C17	1.6 (2)
C4—C5—C6—C7	−177.55 (18)	C14—C15—C16—C17	176.85 (16)
C1—C6—C7—C8	0.51 (17)	C15—C16—C17—C18	−2.0 (3)
C5—C6—C7—C8	179.17 (17)	C21—O4—C18—C19	−178.81 (19)
C1—C6—C7—C9	−178.39 (17)	C21—O4—C18—C17	1.0 (3)
C5—C6—C7—C9	0.3 (3)	C16—C17—C18—O4	−178.54 (16)
C6—C7—C8—N1	0.14 (17)	C16—C17—C18—C19	1.2 (3)
C9—C7—C8—N1	179.02 (17)	O4—C18—C19—C20	179.64 (15)
C6—C7—C8—C22	−176.54 (16)	C17—C18—C19—C20	−0.2 (2)
C9—C7—C8—C22	2.3 (3)	C18—C19—C20—C15	−0.2 (2)
C1—N1—C8—C7	−0.75 (17)	C16—C15—C20—C19	−0.4 (2)
C10—N1—C8—C7	179.66 (13)	C14—C15—C20—C19	−175.62 (14)
C1—N1—C8—C22	176.17 (14)	C7—C8—C22—O1	−6.8 (3)
C10—N1—C8—C22	−3.4 (2)	N1—C8—C22—O1	176.90 (19)
C1—N1—C10—C11	72.45 (17)		

### Hydrogen-bond geometry (Å, º)

Cg1 and Cg2 are the centroids of the N1/C1/C6–C8 and C1–C6 rings, respectively.

**Table:**

*D*—H···*A*	*D*—H	H···*A*	*D*···*A*	*D*—H···*A*
C9—H9*A*···O1	0.96	2.53	3.033 (3)	113
C14—H14···O2	0.93	2.41	2.789 (2)	104
C10—H10*B*···O2^i^	0.97	2.51	3.480 (2)	173
C22—H22···O2^i^	0.93	2.49	3.409 (3)	171
C17—H17···*Cg*1^ii^	0.93	2.76	3.573 (2)	146
C21—H21*A*···*Cg*2^ii^	0.96	2.84	3.635 (3)	140

Symmetry codes: (i) −*x*+3/2, *y*+1/2, −*z*+1/2; (ii) *x*−1/2, −*y*+1/2, *z*−1/2.

## References

[bb1] Andreani, A., Granaiola, M., Leoni, A., Locatelli, A., Morigi, R., Rambaldi, M., Giorgi, G., Salvini, L. & Garaliene, V. (2001). *Anticancer Drug. Des.* **16**, 167–174. 11962514

[bb2] Bruker (2001). *SMART*, *SADABS* and *SAINT* Bruker AXS Inc., Madison, Wisconsin, USA.

[bb3] Farrugia, L. J. (2012). *J. Appl. Cryst.* **45**, 849–854.

[bb4] Gans, J. D. & Shalloway, D. (2001). *J. Mol. Graph. Model.* **19**, 557–559. 10.1016/s1093-3263(01)00090-0 11552684

[bb5] Grinev, A. N., Shevdov, V. L., Krichevskii, E. S., Romanova, O. B., Altukkhova, L. B., Kurilo, G. N., Andreeva, N. I., Golovina, S. M. & Mashkovskii, M. D. (1984). *Khim. Farm. Zh.* **18**, 159–163.

[bb6] Kaushik, N. K., Kaushik, N., Attri, P., Kumar, N., Kim, C. H., Verma, A. K. & Choi, E. H. (2013). *Molecules*, **18**, 6620–6662. 10.3390/molecules18066620 PMC6270133 23743888

[bb7] Rodriguez, J. G., Temprano, F., Esteban-Calderon, C., Martinez-Ripoll, M. & Garcia-Blanco, S. (1985). *Tetrahedron*, **41**, 3813–3823.

[bb8] Selvanayagam, S., Sridhar, B., Ravikumar, K., Kathiravan, S. & Raghunathan, R. (2008). *Acta Cryst.* E**64**, o1163. 10.1107/S160053680801547X PMC2961622 21202671

[bb9] Sheldrick, G. M. (2008). *Acta Cryst.* A**64**, 112–122. 10.1107/S0108767307043930 18156677

[bb10] Singh, U. P., Sarma, B. K., Mishra, P. K. & Ray, A. B. (2000). *Folia Microbiol. (Praha)*, **45**, 173–176. 10.1007/BF02817419 11271828

[bb11] Spek, A. L. (2009). *Acta Cryst.* D**65**, 148–155. 10.1107/S090744490804362X PMC2631630 19171970

